# Diversity of *Bathyarchaeia* viruses in metagenomes and virus-encoded CRISPR system components

**DOI:** 10.1093/ismeco/ycad011

**Published:** 2024-01-10

**Authors:** Changhai Duan, Yang Liu, Ying Liu, Lirui Liu, Mingwei Cai, Rui Zhang, Qinglu Zeng, Eugene V Koonin, Mart Krupovic, Meng Li

**Affiliations:** SZU-HKUST Joint PhD Program in Marine Environmental Science, Shenzhen University, Shenzhen 518060, China; Archaeal Biology Center, Institute for Advanced Study, Shenzhen University, Shenzhen 518060, China; Shenzhen Key Laboratory of Marine Microbiome Engineering, Institute for Advanced Study, Shenzhen University, Shenzhen 518060, China; Department of Ocean Science, The Hong Kong University of Science and Technology, Hong Kong 999077, China; Archaeal Biology Center, Institute for Advanced Study, Shenzhen University, Shenzhen 518060, China; Shenzhen Key Laboratory of Marine Microbiome Engineering, Institute for Advanced Study, Shenzhen University, Shenzhen 518060, China; Institut Pasteur, Université Paris Cité, Archaeal Virology Unit, Paris 75015, France; Archaeal Biology Center, Institute for Advanced Study, Shenzhen University, Shenzhen 518060, China; Shenzhen Key Laboratory of Marine Microbiome Engineering, Institute for Advanced Study, Shenzhen University, Shenzhen 518060, China; Archaeal Biology Center, Institute for Advanced Study, Shenzhen University, Shenzhen 518060, China; Shenzhen Key Laboratory of Marine Microbiome Engineering, Institute for Advanced Study, Shenzhen University, Shenzhen 518060, China; Archaeal Biology Center, Institute for Advanced Study, Shenzhen University, Shenzhen 518060, China; Shenzhen Key Laboratory of Marine Microbiome Engineering, Institute for Advanced Study, Shenzhen University, Shenzhen 518060, China; Department of Ocean Science, The Hong Kong University of Science and Technology, Hong Kong 999077, China; National Center for Biotechnology Information, National Library of Medicine, National Institutes of Health, Bethesda, MD 20894, USA; Institut Pasteur, Université Paris Cité, Archaeal Virology Unit, Paris 75015, France; SZU-HKUST Joint PhD Program in Marine Environmental Science, Shenzhen University, Shenzhen 518060, China; Archaeal Biology Center, Institute for Advanced Study, Shenzhen University, Shenzhen 518060, China; Shenzhen Key Laboratory of Marine Microbiome Engineering, Institute for Advanced Study, Shenzhen University, Shenzhen 518060, China

**Keywords:** Bathyarchaeia, virus, Type IV-B CRISPR-Cas system, Cas4 protein

## Abstract

*Bathyarchaeia* represent a class of archaea common and abundant in sedimentary ecosystems. Here we report 56 metagenome-assembled genomes of *Bathyarchaeia* viruses identified in metagenomes from different environments. Gene sharing network and phylogenomic analyses led to the proposal of four virus families, including viruses of the realms *Duplodnaviria* and *Adnaviria*, and archaea-specific spindle-shaped viruses. Genomic analyses uncovered diverse CRISPR elements in these viruses. Viruses of the proposed family “*Fuxiviridae*” harbor an atypical Type IV-B CRISPR-Cas system and a Cas4 protein that might interfere with host immunity. Viruses of the family “*Chiyouviridae*” encode a Cas2-like endonuclease and two mini-CRISPR arrays, one with a repeat identical to that in the host CRISPR array, potentially allowing the virus to recruit the host CRISPR adaptation machinery to acquire spacers that could contribute to competition with other mobile genetic elements or to inhibit host defenses. These findings present an outline of the *Bathyarchaeia* virome and offer a glimpse into their counter-defense mechanisms.

## Introduction

*Bathyarchaeia*, formerly the Miscellaneous Crenarchaeotal Group (MCG), is an archaeal class widespread in marine and freshwater sediments [1-6]. The estimated global abundance of *Bathyarchaeia* reaches up to 2.0–3.9 × 10^28^ cells, representing one of the most abundant groups of archaea on Earth [7]. Genomic analyses suggest that *Bathyarchaeia* lead an acetyl-CoA-centered heterotrophic lifestyle with the potential for acetogenesis [7], methane metabolism [8], and sulfur reduction [9]. *Bathyarchaeia* also encompass a variety of genes encoding carbohydrate-active enzymes [10] and thus likely can unitize various carbohydrates and lignin [11]. The diverse metabolic potential of *Bathyarchaeia* contributes to their predominance in sedimentary environments, rendering them essential players in the global carbon cycle [5, 6, 9, 12].

Viruses, as the most abundant biological agents on the planet [13, 14], have a major impact on the composition and activity of microbial communities [15-18]. Bacteria and archaea evolved enormously versatile repertoires of antivirus immune systems. In particular, nearly all archaea and many bacteria encode CRISPR-Cas, the prokaryotic adaptive immunity systems [19]. The CRISPR-Cas system selectively acquires foreign DNA fragments (protospacers) and stores them as spacers in the CRISPR array, which is expressed to produce CRISPR (cr) RNAs that serve as guides recognizing the DNA or RNA target and recruiting CRISPR effector nucleases [20]. The CRISPR-Cas systems provide the most reliable basis for viral host prediction by linking host spacers to the cognate virus protospacers [21].

In response to the host defenses, viruses infecting bacteria and archaea evolved a broad repertoire of counter-defense mechanisms to evade immunity [22-24], engaging in the perennial arms race. In particular, many viruses encode diverse anti-CRISPR proteins (Acrs) that specifically target different CRISPR-Cas subtypes [25, 26]. These Acrs function by directly interacting with various components of the CRISPR-Cas system [27-29] or modulating the levels of cyclic oligoadenylate [30], thereby suppressing the functionality of the CRISPR-Cas system.

To date, only a limited number of archaeal viruses have been isolated by traditional cultivation-dependent methods [31-34]. In recent years, an increasing diversity of archaeal viruses has been uncovered through metagenomic data mining [35], including viruses associated with Asgard archaea [36-38], methanogenic archaea [39], methanotrophic ANME-1 archaea [40], ammonia-oxidizing archaea [41-44], and marine Group II *Euryarchaeota* (Poseidoniales) [45]. Although viruses linked to *Bathyarchaeia* have been reported [46-49], their gene content, diversity, classification, and virus–host interactions have not comprehensively assessed, warranting further investigation. Here, we describe the results of metagenomic analysis revealing a substantial diversity of viruses associated with *Bathyarchaeia*, some of which encode CRISPR-Cas system components and predicted Acr could potentially interact with host immunity systems.

## Results and Discussion

### Discovery of viruses associated with *Bathyarchaeia*

**Figure 1:**
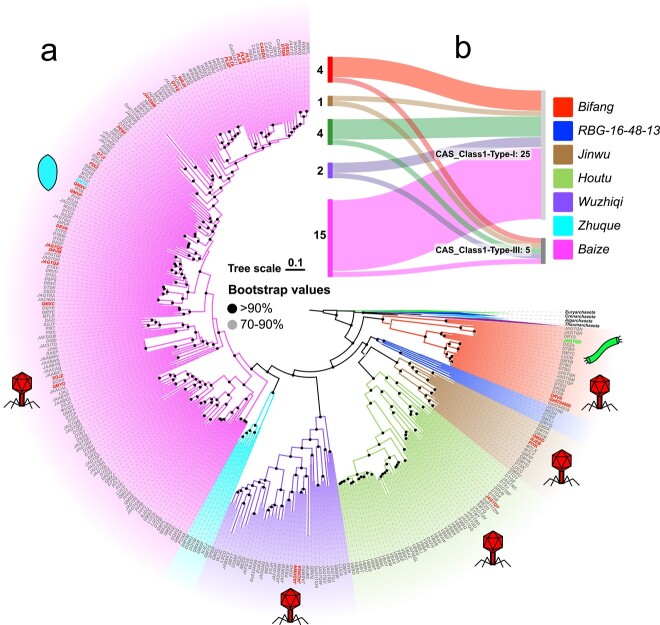
Distribution of identified viruses across the evolutionary tree of the *Bathyarchaeia*; (A) maximum likelihood tree of *Bathyarchaeia* was reconstructed based on the modified set of 51 marker genes; the outgroup was taken from Ren *et al*. [116] and includes representatives of the phyla *Euryarchaeota*, *Crenarchaeota*, *Aigarchaeota*, and *Thaumarchaeota*; *Bathyarchaeia* orders are highlighted in different background colors; viruses identified in this study are indicated with colored symbols denoting the respective virion architectures, and their corresponding hosts are highlighted with the same color; red icosahedron represents viruses in the realm *Duplodnaviria*, green rod represents viruses in the realm *Adnaviria*, and blue spindle represents the spindle-shaped virus; (B) subtypes of CRISPR-Cas systems distributed in different *Bathyarchaeia* orders: Bifang, Bifangarchaeales; Jinwu, Jinwuousiales; Houtu, Houtuarculales; Wuzhiqi, Wuzhiqiibiales; Zhuque, Zhuquarculales; Baize, Baizomonadales; only high-quality MAGs were selected.

**Figure 2:**
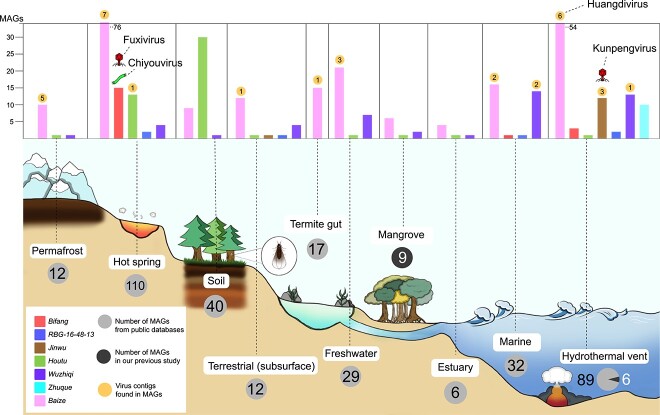
Ecological distribution of *Bathyarchaeia* and their viruses; the number of *Bathyarchaeia* orders in different types of habitats are shown by the bar chart, with the number of detected viral sequences discovered in the genomes shown in the circles above the corresponding bars; viruses identified in this study by matching CRISPR spacers (the distribution of these viruses aligns with their hosts’ habitats) are marked with symbols that denote their respective virion architectures, placed above their corresponding hosts; MAGs from public databases and from our previous study are denoted by grey and black circles, respectively, with the quantity displayed inside the circles.

In total, 367 metagenome-assembled genomes (MAGs) of *Bathyarchaeia*, including 78 high-quality MAGs (Extended Data Supplementary Table S1), were obtained from our previous results [9, 50] and public databases. Phylogenetic analysis based on a concatenated alignment of an optimized set of 51 marker proteins (see Methods) yielded 7 major clades of *Bathyarchaeia* that mostly correspond to the orders in the latest taxonomy [51] (Fig. 1A and Extended Data Supplementary Fig. S1. The biome distribution survey showed that these *Bathyarchaeia* MAGs covered diverse ecosystems, including hot springs, hydrothermal vents, and mangroves, consistent with a previous 16S rRNA gene-based ecological distribution survey [5]. Moreover, some MAGs of *Bathyarchaeia* were found in freshwater sediments, soil, and the termite guts (Fig. 2). Different *Bathyarchaeia* orders also exhibit environmental preferences. For instance, Bifangarchaeales primarily occur in hot environments, whereas Zhuquarculales are only found in hydrothermal vents. By contrast, Baizomonadales are found in all environments, indicating their strong environmental adaptability.

We subsequently conducted a search for defense systems within *Bathyarchaeia* MAGs, focusing particularly on the well-studied CRISPR-Cas systems. In our analysis, we identified 39 distinct types of defense systems across 367 *Bathyarchaeia* MAGs. The three most widely distributed systems were DNA-modification systems, Phage defense candidate, and CRISPR-Cas systems, found, respectively, in 47.4%, 47.1%, and 22.9% of the genomes (Extended Data Table 1), suggesting these as the primary mechanisms of defense. In addition, commonly recognized defense systems such as restriction modification (found in 65 MAGs), AbiE (abortive infection) (found in 45 MAGs), as well as the recently discovered CBASS (found in 16 MAGs) and viperins (found in 24 MAGs) systems were also identified in a subset of *Bathyarchaeia* MAGs. The wide distribution of these defense systems suggests intense virus–host interactions in *Bathyarchaeia* (Extended Data Table 1). We then delved deeper into the CRISPR-Cas systems in high-quality *Bathyarchaeia* MAGs and identified Type I and Type III CRISPR-*cas* loci in over one-third of the MAGs. In most instances, a bathyarchaeal genome was found to carry a single CRISPR-Cas system of either Type I (17 high quality MAGs) or Type III (1 high quality MAGs). Notably, however, eight MAGs were found to encompass more than one type of CRISPR-Cas systems (Fig. 1B). To identify putative viruses of the *Bathyarchaeia*, a dataset of CRISPR spacers was compiled from all collected MAGs. After automatic screening followed by manual inspection (see Materials and methods), 49 high-confidence CRISPR array from different Bathyarchaeia orders were identified, containing 1602 spacers (Extended Data Supplementary Fig. S2). The spacers were used to search for protospacers in the viral sequences of IMG/VR v3 database [52]. Additionally, a search for potential proviruses in the host genomes was conducted (see Materials and methods). In total, 56 contigs were assigned to *Bathyarchaeia* viruses based on CRISPR spacer matches or unequivocal integration into the host genome. Among these, 54 contigs were found to encode major capsid proteins (MCPs), attesting to their viral nature [53]. Six viral contigs corresponded to complete genomes, as indicated by the presence of terminal repeats (Extended Data Supplementary Table S3).

### Four putative families of *Bathyarchaeia* viruses

**Figure 3:**
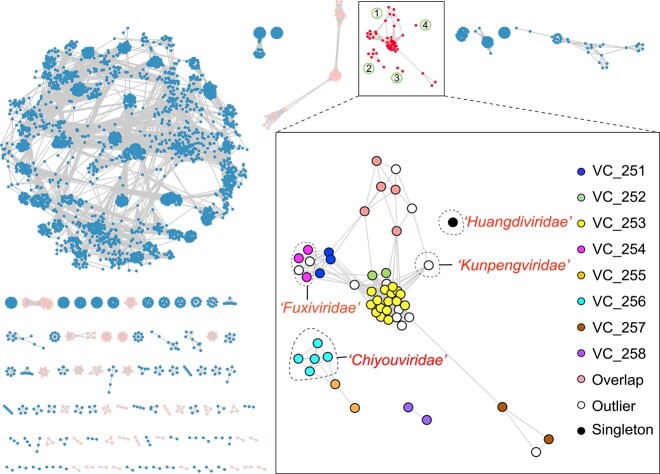
Classification of *Bathyarchaeia* viruses based on the whole-genome protein-sharing network with other prokaryotic viruses; the whole-genome protein-sharing network analysis was constructed using vConTACT2 v.0.11.3 for the taxonomic assignment of 56 *Bathyarchaeia* viral genomes; *Bathyarchaeia* virus clusters are outlined with a rectangle in the complete network; *Bathyarchaeia* viruses are assigned to four distinct groups (numbered within circles), including one large cluster; viral clusters (VCs) are indicated by the colored spheres within the inset; the proposed virus families with complete genomes are separated by dashed lines and appended with the corresponding names; the light pink and light blue clusters outside of the inset represent archaeal and bacterial viruses, respectively; the networks were visualized with Cytoscape v.3.9.1.

Protein sharing network analysis identified four distinct groups of *Bathyarchaeia* viruses, including a large group that consisted of six viral clusters, roughly equivalent to genus-level groups [54] (Fig. 3). Additionally, we carried out a genome-wide sequence similarity comparison and examined the phylogenies of hallmark genes of *Bathyarchaeia* viruses, comparing them with known archaeal viruses (Extended Data Supplementary Figs S3–S5, see online supplementary material for a color version of this figure). Based on the results of these analyses, we identified three distinct types of viruses, encompassing four putative family-level groups. The families “*Fuxiviridae*” and “*Kunpengviridae*” include head-tailed viruses of the class *Caudoviricetes* in the realm *Duplodnaviria*. The family “*Chiyouviridae*” consists of filamentous viruses of the archaea-specific realm *Adnaviria* [55]. The fourth putative family, “*Huangdiviridae*,” with only one representative genome, includes an archaea-specific spindle-shaped virus; the spindle-shaped viruses have not yet been classified at higher taxonomy ranks (Fig. 3).

**Figure 4:**
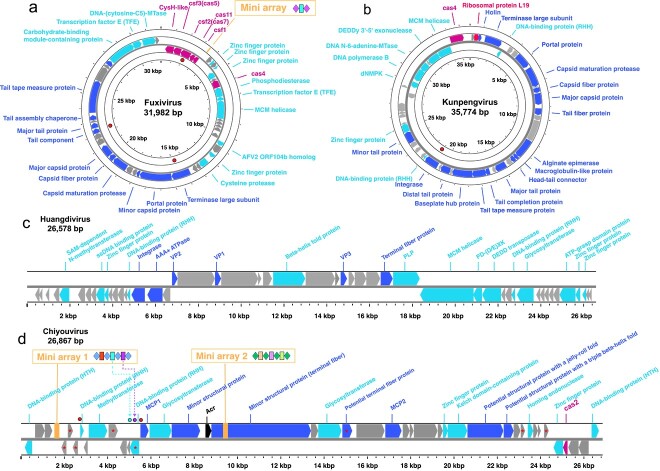
Maps of complete genomes of *Bathyarchaeia* viruses; (A) Genome map of Fuxivirus; (B) genome map of Kunpengvirus; (C) genome map of Huangdivirus; (D) genome map of Chiyouvirus; genes annotated by HHblits with a probability greater than 95% are shown as different colors; genes related to Type IV B CRISPR-Cas system, Cas4, and Cas2 are indicated in rose pink, mini-CRISPR array in vibrant orange, genes specific to viruses in dark blue, predicted Acr in black, other annotated genes in light blue; the positions of targeted protospacers are indicated with red circle; the organization of CRISPR mini-arrays is shown above the genome maps; in Chiyouvirus CRISPR Array 1, the self-targeting spacers are highlighted in light blue and purple; their corresponding target sites on the genome are marked with circles in the same color; transmembrane proteins of Chiyouvirus predicted using CCTOP [117] server are indicated with red asterisks; fdetailed gene annotations are in Supplementary Table S4.

The proposed family “*Fuxiviridae*” is represented by three nearly identical complete genomes (Fuxivirus) from hot springs (Fig. 2) that encompass protospacers targeted by Type I-A CRISPR spacers from the *Bathyarchaeia* order Bifangarchaeales (Figs 1 and 4, Extended Data Supplementary Table S2). Fuxivirus has a smaller genome compared to the typical size of archaeal viruses of the class *Caudoviricetes* (median size of 54.3 kb, n = 44), with a length of 31 982 bp (Fig. 4A). Fuxivirus encodes all the hallmark proteins of *Caudoviricetes*, namely, a HK97-like MCP (gene Fuxivirus_34 and Extended Data Supplementary Fig. S3B), a portal protein (gene Fuxivirus_28), a terminase large subunit (LSU) (gene Fuxivirus_27), a tail tube protein (gene Fuxivirus_38), and several other tail components (Fig. 4A and Extended Data Supplementary Table S4), which are similar to those of the previously characterized archaeal tailed viruses [56]. In addition to the viral hallmark genes, Fuxivirus encodes several putative DNA-binding proteins, such as predicted transcription factors containing Zn-finger and winged-helix-like domains (Fig. 4A and Extended Data Supplementary Table S4) that likely regulate viral or host gene expression [57]. Notably, Fuxivirus also encodes CRISPR-Cas system components, namely, a Type IV *cas* gene cluster (genes Fuxivirus_1 to Fuxivirus_5) with a mini CRISPR array, and a Cas4-like protein (gene Fuxivirus_12) (Fig. 4A).

The proposed family “*Kunpengviridae*” was detected in hydrothermal vents (Fig. 2) and includes one complete viral genome (Kunpengvirus), that is targeted by a single spacer (100% match) from *Bathyarchaeia* sp. QMXD of the order Jinwuousiales (Figs 1 and 4B, Extended Data Supplementary Table S2). Kunpengvirus encodes the hallmark capsid morphogenesis proteins of *Caudoviricetes*, as well as a suite of tail proteins including the baseplate protein (Fig. 4B and Extended Data Supplementary Table S4). Kunpengvirus also encodes an integrase (gene Kenpengvirus_28), suggesting that it can integrate into the host genome as a provirus. In addition, this virus encompasses genes for a deoxynucleoside monophosphate kinase (gene Kenpengvirus_41), an MCM-like helicase (gene Kenpengvirus_46) and a family B DNA polymerase (gene Kenpengvirus_42) (Fig. 4B and Extended Data Supplementary Table S4), indicative of (at least, partially) autonomous genome replication [58]. Notably, unlike in other tailed viruses, the proofreading DEDDy 3′-5′ exonuclease (gene Kenpengvirus_45) and the family B DNA polymerase domains are encoded by two distinct genes. Additionally, Kunpengvirus encodes a homolog of ribosomal protein bL19 (gene Kunpengvirus_2), which is typically present in bacteria and eukaryotes (chloroplasts and mitochondria) but not in archaea, except for *Candidatus Aenigmarchaeota*. The bL19 is located at the 30S–50S ribosomal subunit interface and is thought to contribute to the structure and function of the aminoacyl-tRNA binding site [59]. A number of ribosomal proteins, including bL19, were previously identified in bacterial viruses [60], but the only ribosomal protein so far detected in archaeal viruses had been L21e [56]. The presence of bL19 in Kunpengvirus suggests that modification of the host translation apparatus by archaeal viruses is more common than currently recognized. Similar to the Fuxivirus, Kunpengvirus also encodes a Cas4-like endonuclease (Fig. 4B and gene Kenpengvirus_47).

A unique viral genome (Huangdivirus) representing the proposed family “*Huangdiviridae*” was identified as an apparent provirus in *Bathyarchaeia* sp. QMYA of the order Baizomonadales from a deep-sea hydrothermal vent (Figs 1 and 2). Sensitive sequence comparison using HHblits identified three virus-encoded structural proteins (VPs) VP1–3 (Fig. 4C and Extended Data Supplementary Table S4), homologous to the structural proteins of archaeal spindle-shaped viruses of the family *Fuselloviridae*, as well as an AAA+ ATPase (gene Huangdivirus_17, HHblits best hit to ATV ATPase, with 99.8% probability). Structural predictions for VP1 (gene Huangdivirus_21) and VP3 (gene Huangdivirus_28) indicated that both proteins contain two hydrophobic α-helices connected by a short turn (Extended Data Supplementary Fig. S4A) resembling the typical structure of the MCPs of spindle-shaped viruses [61]. Huangdivirus VP2 (gene Huangdivirus_19) is most closely related to the viral DNA-binding protein VP2 of Sulfolobus spindle-shaped virus 1 (SSV1) [62] (HHblits probability of 99.49%). As in the case of SSV1 [62], we detected the consensus glycosylation motifs (N-X-S/T) in Huangdivirus VP1 and VP3, which may be glycosylated by the virus-encoded glycosyltransferase (gene Huangdivirus_40 and Extended Data Supplementary Fig. S5B). Capsid protein glycosylation is thought to contribute to the virion stability, particularly for viruses infecting hyperthermophilic hosts [62]. Similar to fuselloviruses infecting hyperthermophilic archaea of the order Sulfolobales [63], Huangdivirus encodes a putative integrase of the tyrosine recombinase superfamily (gene Huangdivirus_16; Fig. 4C), which is likely to be responsible for viral DNA integration into the host chromosome. Notably, Huangdivirus encodes a predicted polysaccharide lyase (Fig. 3C and gene Huangdivirus_33) that might be involved in the break-down of the S-layer polysaccharides [64, 65] of host cell wall during infection.

“*Chiyouviridae*” is a potential new family of filamentous viruses in the order *Ligamenvirales* (class *Tokiviricetes*, realm *Adnaviria*). “*Chiyouviridae*” was detected in a hot spring and represented by one complete viral genome (Chiyouvirus; Fig. 2) targeted by two spacers of *Bathyarchaeia* sp. JAGTQC in the order Bifangarchaeales (Figs 1 and 4D). Phylogenomic analysis of all available *Tokiviricetes* [66] genomes recapitulated the previously established relationships and showed that Chiyouvirus forms a separate clade within the order *Ligamenvirales*, most closely related to the families *Rudiviridae* and *Ungulaviridae* (Extended Data Supplementary Fig. S5A). Whole proteome comparison showed <50% average amino acid identity (AAI) between protein homologs from Chiyouvirus and members of other viral families, with the highest AAI (46%) with the genus *Icerudivirus* of the family *Rudiviridae* (Extended Data Supplementary Fig. S5B). Similar to all members of the families *Ungulaviridae* and *Lipothrixviridae* but only some members of the family *Rudiviridae* [66], Chiyouvirus encodes two MCPs (genes Chiyouvirus_14 and Chiyouvirus_23), each comprising an alpha-helix bundle (Fig. 4D and Extended Data Supplementary Fig. S5C). In rudiviruses, unlike other members of the realm *Adnaviria*, the second MCP paralog, when present, is not incorporated into virions [67]. Thus, it remains unclear whether both Chiyouvirus MCPs are involved in virion formation. In addition, Chiyouvirus encodes a large minor structural protein (Fig. 4D and gene Chiyouvirus_18), a homolog of SIRV2 P1070, which is thought to be involved in the formation of the virion terminal filaments that are responsible for host recognition [68, 69]. Predictions for transmembrane proteins indicated that Chiyouvirus encodes eight potential transmembrane proteins (Fig. 4D) suggesting that it is a membrane-enveloped filamentous virus, similar to the adnaviruses in the families *Lipothrixviridae*, *Ungulaviridae*, and *Tristromaviridae* [70].

### CRISPR-Cas systems and potential counter defense mechanisms in *Bathyarchaeia* viruses

We explored the Type IV CRISPR-Cas system encoded by “*Fuxiviridae*” in greater detail, including phylogenetic analysis and gene locus comparison. The viral CRISPR-*cas* locus encodes all typical components of the Type IV CRISPR-Cas effector module (Fig. 4A and Extended Data Supplementary Table S4), in particular, the signature protein Csf1 (gene Fuxivirus_5), the apparent LSU of the effector complex, but no adaptation module. Phylogenetic analysis of Cas proteins, including Csf2 (Cas7) (gene Fuxivirus_3), the most conserved protein in the Type IV systems [71], showed that CRISPR-Cas system of “*Fuxiviridae*” belongs to the IV-B subtype (Extended Data Supplementary Fig. S6). Additionally, a CysH-like protein (gene Fuxivirus_1), which is tightly associated with the Type IV-B systems, is encoded in the virus genome adjacent to Csf3 (Cas5) (gene Fuxivirus_2; Fig. 4A). Phylogenetic analysis showed that Fuxivirus CysH-like protein does not belong to the viral CysH branch but is rather associated with CysH-like proteins from other Type IV-B systems (Extended Data Supplementary Fig. S6C). Previously, Type IV CRISPR-Cas systems have been observed in plasmids and prophages [72, 73] as well as several lytic phages [74], but not in archaeal viruses.

Most Type IV-B systems lack both the adaptation module and a CRISPR array, but the Type IV-B CRISPR-*cas* locus in the Fuxivirus genome contains a CRISPR mini-array that consists of two repeats and a spacer (Fig. 4A). A Type IV-B system has been reported to form a filamentous RNP complex, which predominantly assembles on non-CRISPR RNAs, without apparent sequence specificity, suggesting a function distinct from adaptive immunity [75]. However, given the presence of the mini-array, we hypothesize that the Fuxivirus Type IV-B complex could incorporate spacers, conceivably, by recruiting the host adaptation machinery, and utilize a virus-encoded crRNA to target host DNA or other coinfecting mobile genetic elements (MGEs), thus, being potentially involved in evading host immunity and/or inter-MGE conflicts [76]. However, no full-length protospacer matches for the Fuxivirus mini-array spacer sequence were found in either the host or other MGEs, so that further validation of the counter-defense function of the Fuxivirus Type IV-B CRISPR-Cas system is needed.

Although we could not detect a complete CRISPR-Cas system in the partial genome of the inferred Fuxivirus host, *Bathyarchaeia* sp. DRVA contains two CRISPR arrays and two *cas3* gene that are not adjacent to the CRISPR arrays. Notably, a close relative of *Bathyarchaeia* sp. DRVA, *Bathyarchaeia* sp. JAGTQM, from the same order Bifangarchaeales, contains complete Type I-A and III-D CRISPR-Cas systems and was found to share identical CRISPR repeat and a closely similar Cas3 protein (84.03% identity, 100% coverage) with *Bathyarchaeia* sp. DRVA. These observations suggest the presence of a CRISPR-Cas system(s) in the DRVA genome as well. Notably, we found that the *cas7* gene of Fuxivirus was targeted by a host spacer (Fig. 4A), suggesting that the virus-encoded Type IV-B system, and with it, possibly, the virus reproduction, can be inhibited by the host CRISPR-Cas systems.

The Cas4 homologs encoded by “*Fuxiviridae*” (gene Fuxivirus_12) and “*Kunpengviridae*” (gene Kenpengvirus_47) could represent an additional counter-defense factor. Cas4 is a P-D/ExK family nuclease that is a common component of CRISPR-Cas systems that assists Cas1–Cas2 integration complexes in the acquisition of CRISPR spacers [77], but many Cas4 homologs are encoded outside CRISPR-*cas* loci [78]. Phylogenetic analysis showed that these *Bathyarchaeia* viral Cas4-like proteins are most closely related to Cas4 homologs encoded by Sulfolobus-infecting rudiviruses (Extended Data Supplementary Fig. S6B), suggesting the possibility of horizontal gene transfer between unrelated archaeal viruses. Multiple sequence alignment confirmed that two Cas4 homologs encoded by “*Fuxiviridae*” and “*Kunpengviridae*” share nearly all conserved amino acids with the rudivirus SIRV2-encoded Cas4 homolog and are thus predicted to be active nucleases (Extended Data Supplementary Fig. S7). Previous studies have demonstrated that overexpression of the SIRV2-encoded Cas4 in the archaeal host substantially reduced the efficiency of exogenous spacers acquisition by the host CRISPR-Cas system [79]. Thus, the Fuxivirus and Kunpengvirus Cas4 might inhibit spacer acquisition by the CRISPR systems of Bathyarchaeia hosts. However, involvement of this nuclease in the virus genome replication cannot be ruled out either [80].

We identified two mini-arrays in Chiyouvirus genome, each containing four repeats and three spacers (Fig. 4D), and the repeats in Chiyouvirus mini-array 1 are identical to the repeats in the host CRISPR array. Thus, the pre-crRNA transcribed from this viral mini-array is predicted to be processed by the host CRISPR-Cas system and the mature viral crRNA would remain bound by the host effector complex [72]. Furthermore, the virus could potentially recruit the host CRISPR adaptation machinery to incorporate additional spacers into the mini-array and employ the respective crRNAs in inter-MGE competition, as demonstrated for some bacterial and archaeal micro-array carrying viruses [72, 76], or for abrogation of host defenses. However, intriguingly, in the Chiyouvirus mini-array 1, we identified two self-targeting spacers, both with a 100% match to the corresponding protospacers (Fig. 4D). The role of these self-targeting spacers remains unclear. For the spacers in the Chiyouvirus mini-array 2, no potential targets were identified.

In addition to the mini-arrays, Chiyouvirus encodes a homolog of Cas2 nuclease (gene Chiyouvirus_39), with a conserved Mg^2+^ binding site (Extended Data Supplementary Fig. S8), which is an essential structural subunit of the adaptation complex in CRISPR-Cas systems. The virus-encoded Cas2 homolog might be a dominant negative inhibitor of spacer acquisition by the host CRISPR-Cas system.

Additionally, we attempted to predict Acrs among the *Bathyarchaeia* viral proteins by using a recently developed deep learning method [81]. We found that the structural model of a predicted Chiyouvirus Acr protein (gene Chiyouvirus_17) was significantly similar to the N-terminal domain of AcrIF24 (7DTR, chain A) (Extended Data Supplementary Fig. S9), which inhibits the activity of a Type I-F CRISPR-Cas system by forming a dimer and inducing the dimerization of the Csy complex, blocking the hybridization of target DNA to crRNA [82].

Finally, in addition to the components of CRISPR-Cas systems, Fuxivirus and Kunpengvirus encode stand-alone DNA methyltransferases (genes Fuxivirus_47 and Kenpengvirus_43) that could provide protection against the host restriction-modification systems.

To conclude, in this work, we identify four distinct groups of *Bathyarchaeia* viruses that can be expected to become new viral families. Notably, these viruses encode various components of CRISPR-Cas systems that could interfere with the host CRISPR-Cas immunity and/or mediate inter-virus conflicts. Thus, this study provides a glimpse into the virome of a widespread, comparatively abundant but poorly characterized class of archaea and the virus–host interactions in these organisms.

## Material and methods

### *Bathyarchaeia* genome collection and classification

*Bathyarchaeia* genomes were obtained from our previously reported dataset [9, 50] and the NCBI (https://www.ncbi.nlm.nih.gov) and IMG/M (https://jgi.doe.gov) public database with key words “*Bathyarchaeota*,” “*Bathyarchaeia*,” and “MCG” (miscellaneous *Crenarchaeota* group) (up until 30 June 2021). *Bathyarchaeia* genome classification was based on 51 high-coverage marker gene optimized from GTDB [83] v207 (TIGR01171 and TIGR02389 were excluded due to the low coverage). Marker genes were initially identified with GTDB-Tk v2.1.1 [84] using “identify” method. Only MAGs contain >50% of the marker genes were kept. Sequences were then aligned using MAFFT-LINSI v7.457 [85] and trimmed with TrimAl v1.4 [86] (−gappyout). The maximum likelihood phylogenomic tree for concatenated 51 proteins was constructed with IQ-TREE 2.0.6 [87] (best-fit model LG + F + R13, -B 1000, −alrt 1000). The quality, contamination, GC content, and other sequence information of *Bathyarchaeia* MAGs were assessed by CheckM v1.2.2 (default setting) [88]. For each *Bathyarchaeia* MAG, the 23S, 16S, and 5S rRNA genes were initially identified and extracted using Barrnap 0.9 (https://github.com/tseemann/barrnap). Then followed by a manual curation step involving BLASTN checks against the SILVA 138.1 LSU Reference database and the SILVA 138.1 Small Subunit Reference database (https://arbsilva.de/). The tRNA identification was performed using tRNAscan-SE [89]. Biome information was extracted from the GenBank file or relevant literatures.

To delineate *Bathyarchaeia* orders, the maximum likelihood tree based on 51 proteins was converted to an ultra-metric tree and the relative evolutionary divergence (RED) [90] was calculated. An order was called when its branch length in the ultrametric tree corresponded to a RED value within the range of 0.6 ± 0.1.

### *Bathyarchaeia* defense-systems detection and CRISPR array validation

Defense systems (including CRISPR-Cas systems) in *Bathyarchaeia* MAGs were detected using PADLOC [91] (v2) and DefenseFinder [92]. Subtypes of detected CRISPR-Cas systems were classified using CRISPRCasTyper [93]. CRISPR array in *Bathyarchaeia* MAGs were searched with MinCED [94] (Default setting). To remove ambiguous CRISPR array that might not belong to *Bathyarchaeia*, all CRISPR array-containing contigs encoding proteins were searched against the nr database (2022–09) using Diamond [95] (2.1.6) BLASTP (−evalue 1e-5, —more-sensitive), and only contigs with at least three proteins assigned to *Bathyarchaeia* were retained. The validated CRISPR repeats were clustered with 90% identity using an all-against-all BLASTN [96] search (−evalue 1e-5, word size 7).

### *Bathyarchaeia* virus identification and annotation

We first carried out the CRISPR-based virus-host assignments. Spacers from validated CRISPR array were used as a nucleotide database to compare to viral contigs in the IMG/VR v3 [52] database using BLASTN with parameters (−task blastn-short -evalue 1e-5). Only viral contigs harboring protospacers with 100% coverage and a maximum of one mismatch were considered as *Bathyarchaeia* viruses.

In addition, some viruses might form proviruses that cannot be detected by CRISPR-protospacer search. Therefore, we used VirSorter2 v.2.2.3 [97] to identified potential viral sequences in *Bathyarchaeia* MAGs. Specifically, all the *Bathyarchaeia* contigs were filtered using VirSorter2 (—include-groups dsDNAphage, ssDNA —min-length 5000 —min-score 0.5) and only contigs with identifiable viral structural proteins and at least one ORF identified through a BLASTP search against the nr database as *Bathyarchaeia* were retained. Virial genome completeness estimation and the potential host regions trimming were conducted by CheckV 0.8.1 [98]. Coding sequences were identified using Prokka [99] with parameters (—kingdom viruses —gcode 1).

All the *Bathyarchaeia* viral proteins identified as describe above were annotated with DRAM [100] (viral mode) and HHblits [101] (MSA generated with UniRef30_2020_06 with three interactions, Evalue 1e-6, MSA was compared against the PDB_mmCIF70_14_Apr, SCOPe70_2.07, Pfam-A_v35 and UniProt-SwissProt-viral70_3_Nov_2021 databases with parameters -Z 250 -loc -z 1 -b 1 -B 250 -ssm 2 -sc 1 -seq 1 -dbstrlen 10 000 -norealign -maxres 32 000). All the viral genome maps were visualized using Proksee [102].

### *Bathyarchaeia* viral taxonomy assignment

To determine the taxonomic status of *Bathyarchaeia* viruses, the whole genome network analysis was carried out with vConTACT2 [54] (default parameters) against the Viral RefSeq v207 database as well as archaeal viruses proposed in the latest International Committee on Taxonomy of Viruses Report (VMR_MSL38_v1). The resulting networks were visualized using Cytoscape v3.9.1 [103] with an edge-weighted spring embedded model.

For viruses in the realm *Duplodnaviria*, proteome-scale phylogeny was constructed using the VipTree version 3.4 [104]. The analysis was carried out with archaeal virus families in the realm *Duplodnaviria*, according to the latest ICTV taxonomy classification (25 April 2023). For filamentous viruses, proteome-scale phylogeny was constructed by using VICTOR [105] with all known members of the *Tokiviricetes* class. Structural modeling of viral proteins was performed with AlphaFold2 [106] via ColabFold v1.5.1 [107] with pdb70 template mode.

### *Bathyarchaeia* viral CRISPR-Cas systems classification

All the Cas proteins were manually search with HHblits (see above). To determine the CRISPR-Cas system subtype of ‘*Fuxiviridae*’, phylogenetic tree of all the Cas proteins was constructed with reference sequences. For each of the Cas4, Cas7, Cas5, Cas11, and Csf1 proteins, as well as the phosphoadenosine phosphosulfate reductase (CysH) domain-containing proteins, we applied a consistent analysis procedure. Each protein type was aligned with its respective reference sequences [73, 79] (29 for Cas4, 131 for Cas7, 133 for Cas5, 78 for Cas11, 126 for Csf1, and 322 for CysH, including bona fide CysH and Cas-associated CysH-like proteins recalled from 18 988 public reference complete viral genomes [108]) using MUSCLE 5.1 [109]. The alignments were subsequently trimmed by TrimAl v1.4 (−gappyout for Cas4, Cas5, Cas11, Csf1, and CysH, −gt 0.7 for Cas7), and dropping sequences with >50% gaps. Maximum likelihood trees were then computed for each protein type using IQ-TREE 2.0.6, with the best model selected by model finder [110] (-MFP) varying depending on the proteins (LG + I + G4 for Cas4, VT + R10 for Cas7 and Cas5, VT + R3 for Cas11, WAG+I + G4 for Csf1, and WAG+F + R7 for CysH).

All final phylogenetic trees were visualized by tvBOT [111].

### *Bathyarchaeia* viral CRISPR spacer-protospacer match analysis

CRISPR array in *Bathyarchaeia* viral genome were detected with MinCED (-minNR 1). All the spacers obtained from *Bathyarchaeia* virus were BLASTN search (−task blastn-short) with viral database IMG/VR v3 and plasmid sequences database PLSDB database [112] (v. 2021_06_23_v2) as well as *Bathyarchaeia* MAGs in this study. Direct repeats were searched against CRISPR-CAS++ databases [113] and *Bathyarchaeia* CRISPR repeat dataset with BLASTN (−evalue 0.01).

### Identification of *Bathyarchaeia* viral Acrs

The potential Acrs of *Bathyarchaeia* viruses were first predicted using DeepAcr [81]. The protein structures of predicted Acrs, along with Acrs from reference [114], were modeled using AlphaFold2 by ColabFold v1.5.2 in pdb70 template mode. These potential *Bathyarchaeia* virus Acrs were then used as query models for alignment to the reference Acr models using TM-align [115], and only models with a TM score >0.3 were retained. This initial computational analysis was further supplemented with comprehensive meticulous manual inspection of the alignments and structural models.

### Etymology

“*Fuxiviridae*”: Derived from Fuxi, a legendary figure in Chinese mythology known for his diverse talents and abilities. This alludes to the possibility that the virus has multifaceted counter-defense systems, capable of employing various strategies to evade the host’s immune response.

“*Kunpengviridae*”: Named after Kunpeng, a mythical creature in Chinese mythology known for its transformative abilities. The name alludes to the virus’s ability to integrate into the host genome to form proviruses.

“*Huangdiviridae*”: Named after Huangdi, the legendary Chinese sovereign often associated with important inventions. Given Huangdi’s connections (the host MAG of Huangdivirus in the Baizomonadales order) in Chinese mythology.

“*Chiyouviridae*”: Inspired by Chiyou, a symbol of war and invention in Chinese mythology.

## Supplementary Material

Supplementary_figures_ycad011

Supply_Table_latest_ycad011

## Data Availability

All the *Bathyarchaeia* assembled genomes (*n* = 367) used in this study were collected from publicly available databases including the NCBI (https://www.ncbi.nlm.nih.gov) and IMG/M (https://jgi.doe.gov). The accession number for the MAGs is available in Supplementary Table S1. All the viral contigs (*n* = 56) analyzed in this study were collected from the above *Bathyarchaeia* genomes and publicly available database IMG/VR v3 (https://img.jgi.doe.gov/vr). The GenBank accession number of proposed viral family and datasets generated in this study (i.e. viral contigs, protein files for the viruses, alignments, and tree files) can be accessed at [https://doi.org/10.6084/m9.figshare.24540121.v1].

## Code availability

No custom code was used.
