# Supplementary material for: Diversity of Bathyarchaeia viruses in metagenomes and virus-encoded CRISPR system components
Source: ISME Commun. 2024 Jan 10;4(1):ycad011. doi: 10.1093/ismeco/ycad011 (PMC10848311; doi:10.1093/ismeco/ycad011)
Supplement: Supplementary_figures_ycad011 [file supplementary_figures_ycad011.docx]

**Diversity of Bathyarchaeia viruses in metagenomes and virus-encoded CRISPR system components**

**Running Head:** Viruses of Bathyarchaeia

Changhai Duan^1,2,3,4^, Yang Liu^2,3^, Ying Liu^5^, Lirui Liu^2,3^, Mingwei Cai^2,3^, Rui Zhang^2,3^, Qinglu Zeng^4^, Eugene V. Koonin^6^, Mart Krupovic^5^, Meng Li^1,2,3*^

^1^SZU-HKUST Joint PhD Program in Marine Environmental Science, Shenzhen University, 518060 Shenzhen, China

^2^Archaeal Biology Center, Institute for Advanced Study, Shenzhen University, 518060 Shenzhen, China

^3^Shenzhen Key Laboratory of Marine Microbiome Engineering, Institute for Advanced Study, Shenzhen University, 518060 Shenzhen, China

^4^Department of Ocean Science, The Hong Kong University of Science and Technology, Clear Water Bay, Hong Kong, China

^5^Institut Pasteur, Université Paris Cité , Archaeal Virology Unit, 75015 Paris, France

^6^National Center for Biotechnology Information, National Library of Medicine, Bethesda, MD 20894, USA

***Corresponding author**

Meng Li

Email: limeng848@szu.edu.cn

ORCID: 0000-0001-8675-0758


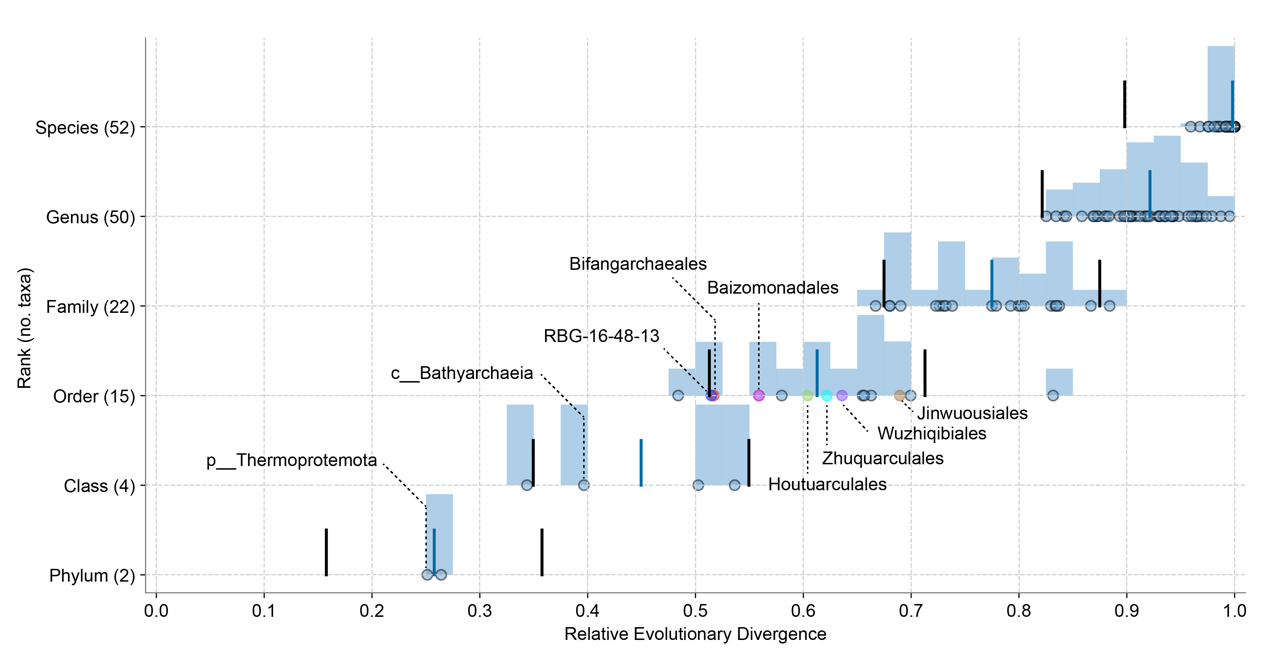


Extended Data Fig. S1: The calculated relative evolutionary divergence (RED) value of each node at order level. Seven order-level units of class Bathyarchaeia are marked in different colors.


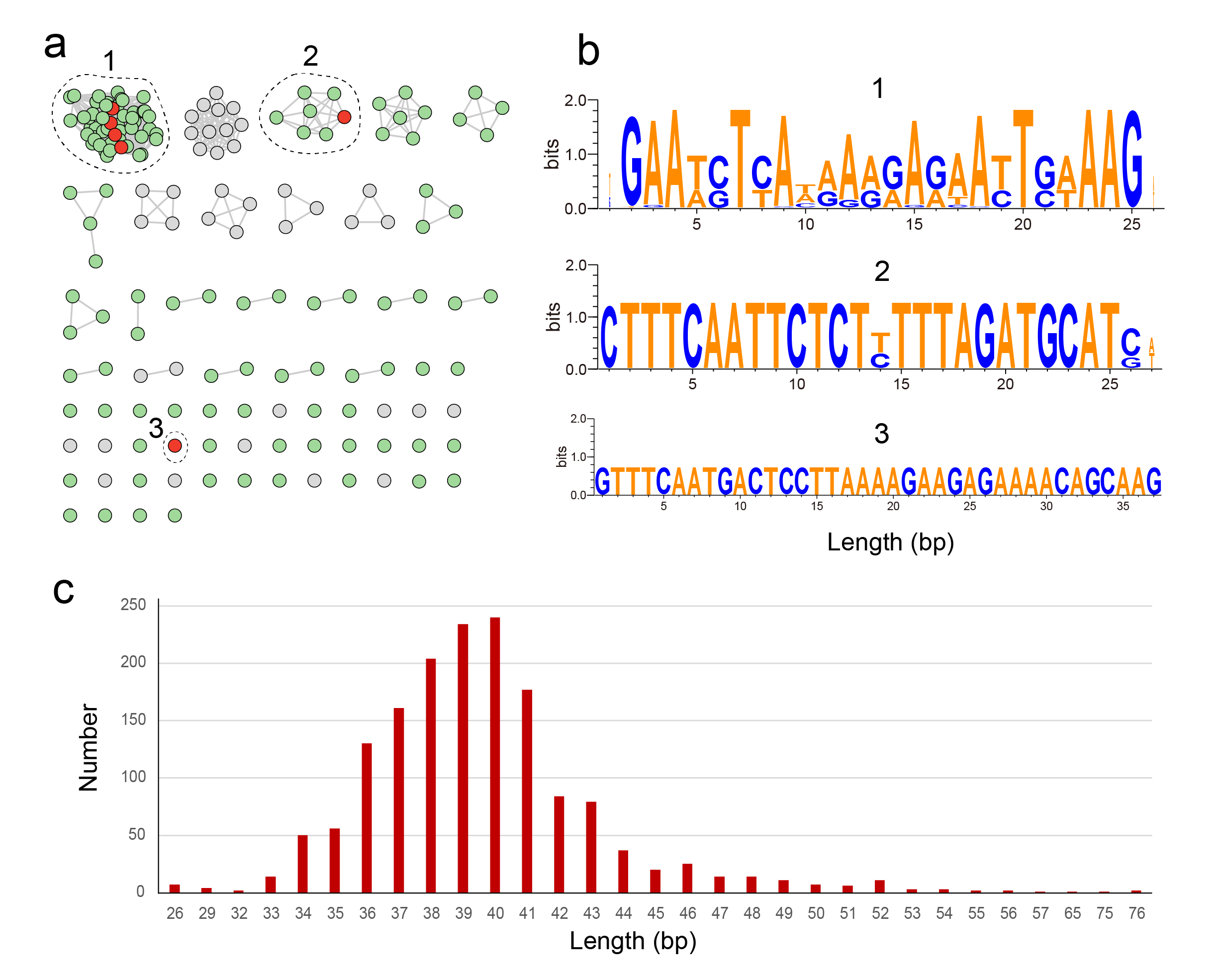


Extended Data Fig. S2: Bathyarchaeia CRISPR Array Overview. a) CRISPR repeats found in Bathyarchaeia MAGs, clustered at 90% sequence identity. Verified repeats are highlight in green, while repeats associated with detected viruses in this study are indicated in red. b) Seq-logo for repeat clusters linked to viruses identified in this research. c) Bathyarchaeia CRISPR spacer length distribution.


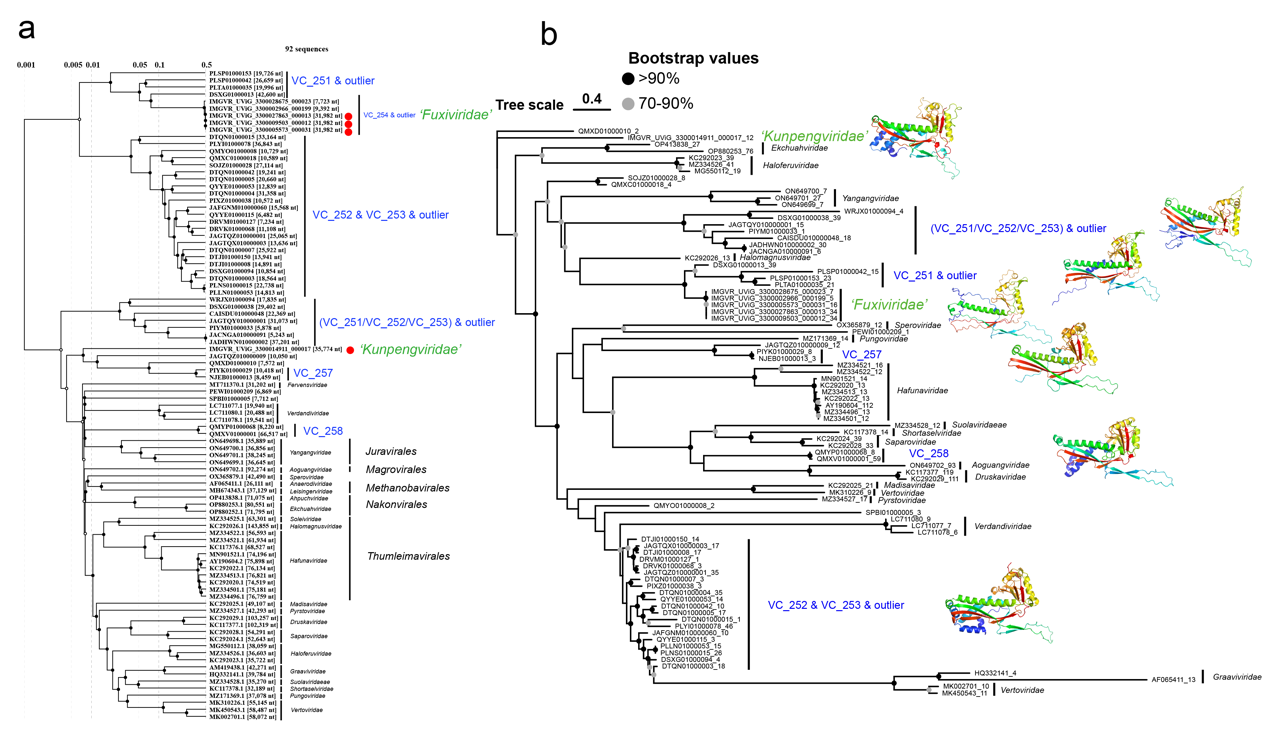


Extended Data Fig. S3: The genome-wide sequence similarity comparison, phylogenetic and modeling of major capsid of Bathyarchaeia viruses in realm *Duplodnaviria*. a) The proteomic tree displays the relationship between Bathyarchaeia viruses and archaea viruses in the realm *Duplodnaviria*, based on genome-wide sequence similarities. Bathyarchaeia virus families are labeled in green, and viruses with complete genomes are marked with a red circle. b) The maximum likelihood tree of major capsid proteins (MCPs) shows the relationship between Bathyarchaeia virus and archaeal virus. Bathyarchaeia virus families are labeled in green, and the 3D structure representing the MCP of each family is displayed next to it.


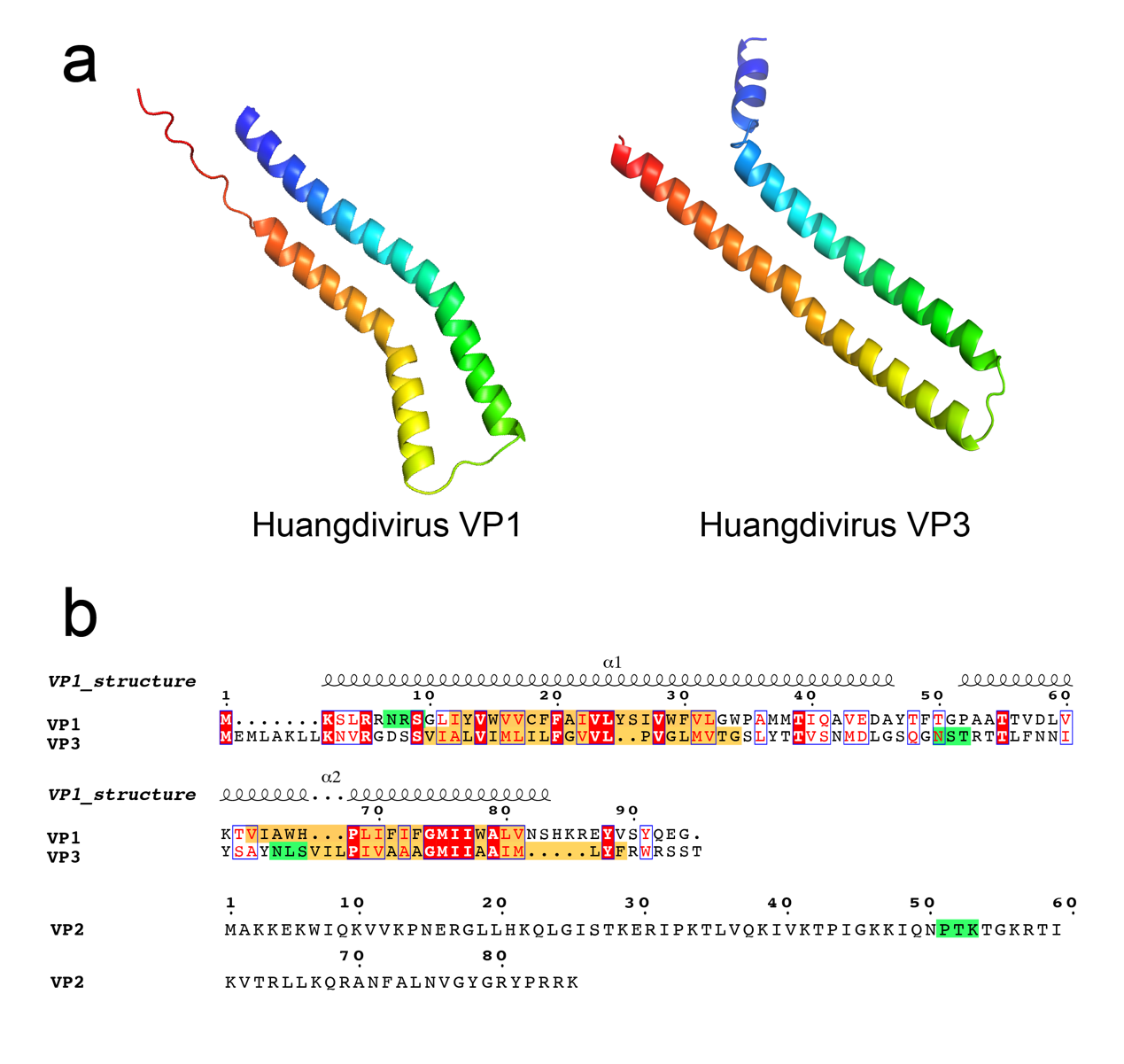


Extended Data Fig. S4: Huangdivirus structural proteins. a) Structural modeling of Huangdivirus capsid proteins VP1 and VP3, colored using a rainbow gradient from N-terminus (blue) to C-terminus (red). b) Sequence analysis of Huangdivirus structural proteins VP1-3, with predicted transmembrane domains highlighted in yellow and theoretical glycosylation consensus motifs (N-X-S/T) shown on a green background.


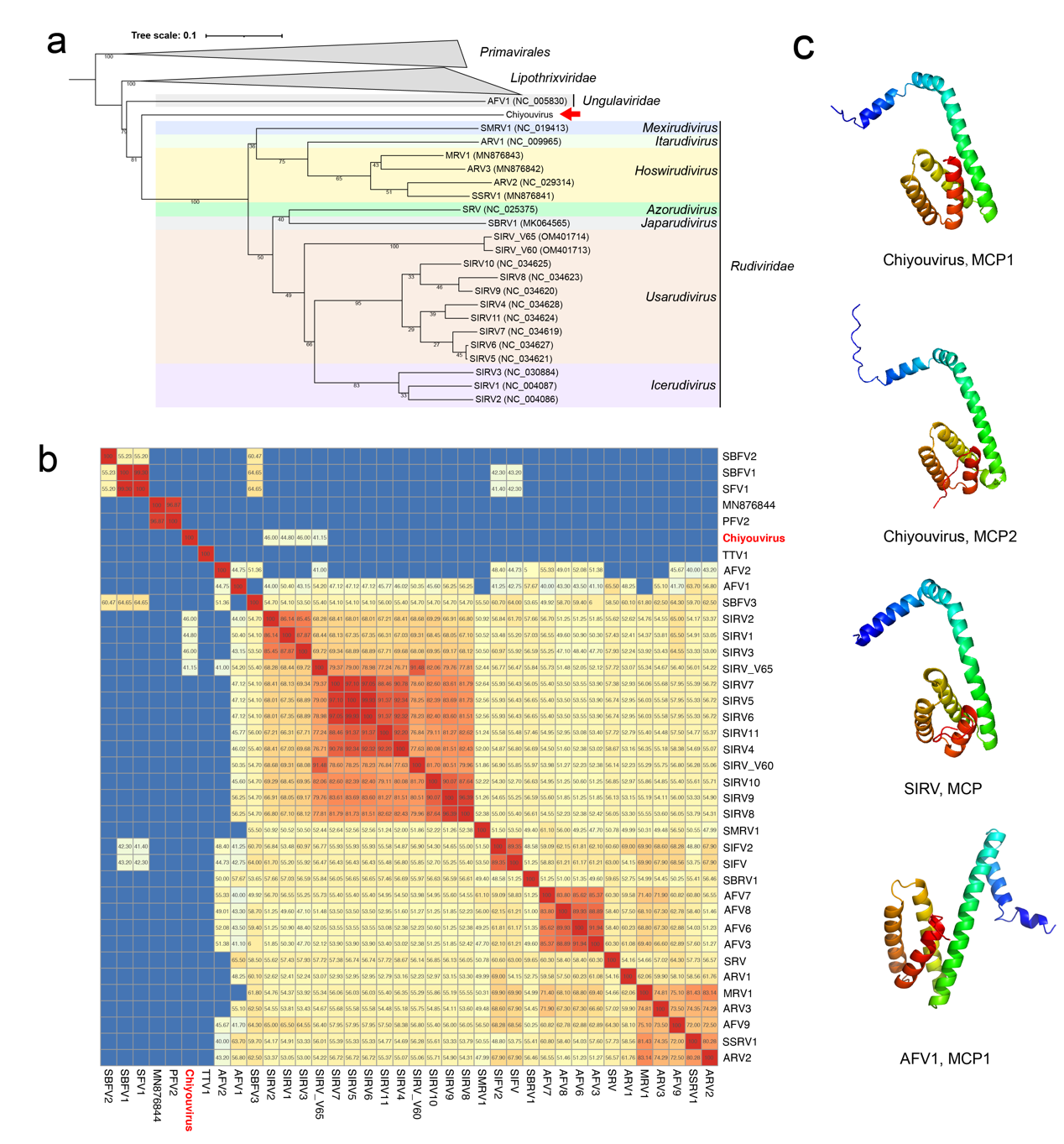


Extended Data Fig. S5: Genomic analysis of Chiyouvirus. a) Phylogenomic tree of Chiyouvirus (red arrow) alongside known members of the *Tokiviricetes* class, based on whole-genome amino acid analysis using VICTOR. Tree is rooted with *Primavirales*, and branch length represents GBDP distance formula D6. Branch support values are indicated with numbers. b) Whole-genome amino acid identity comparison of filamentous viruses in the *Tokiviricetes* class, conducted by EzAAI^1^. Chiyouvirus is highlighted in red. Only AAI values greater than 40% are displayed in the heatmap. c) Predicted structural model comparison of Bathyarchaeial Chiyouvirus major capsid proteins MCP1 and MCP2 with Icerudivirus SIRV (3J9X, chain A) and Captovirus AFV1 (5W7G, chain A) structures. Models are colored using a rainbow gradient from N-terminus (blue) to C-terminus (red).


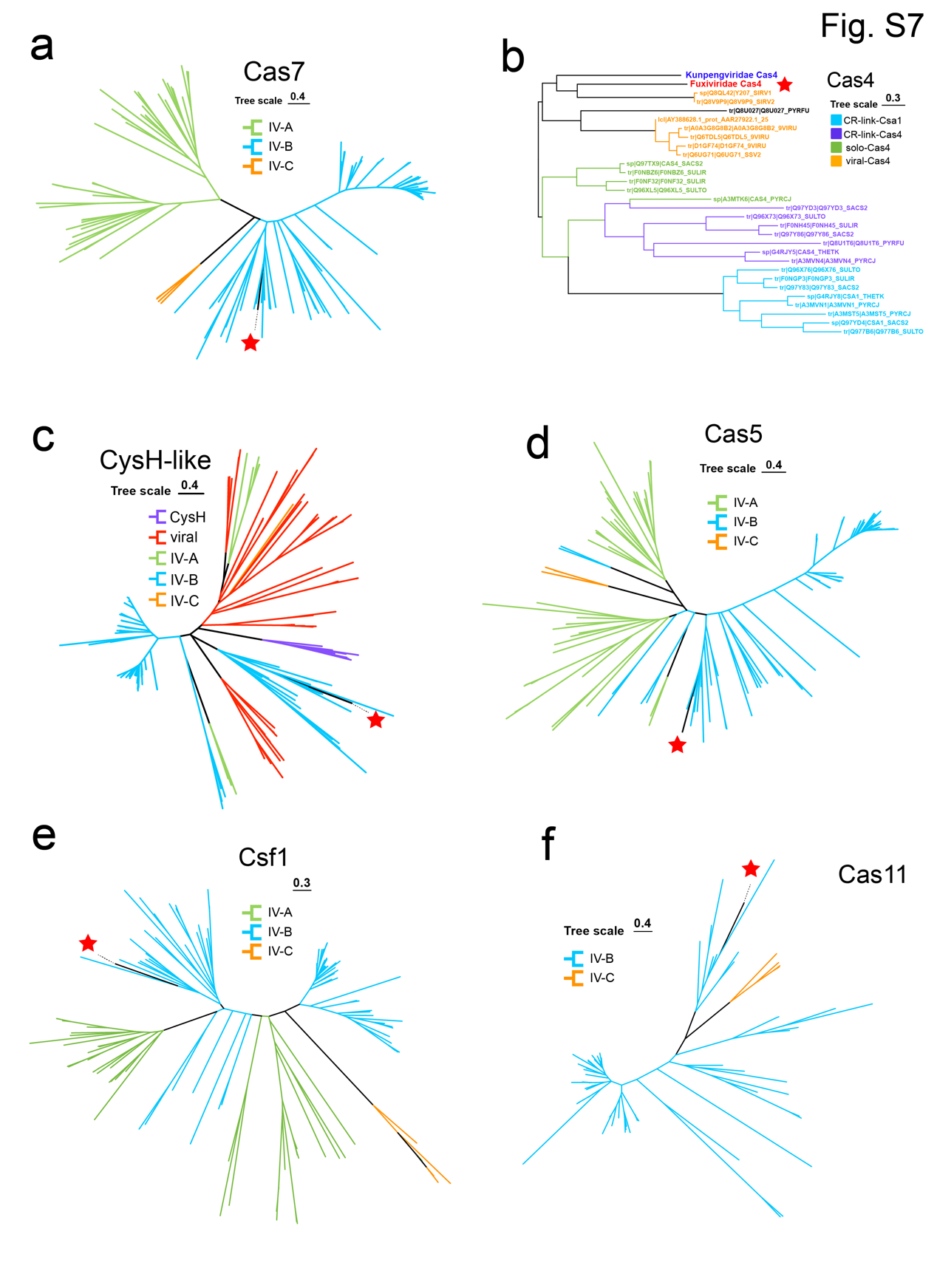


Extended Data Fig. S6: Phylogenetic analysis of proteins in Fuxivirus type IV-B CRISPR-Cas system and Kunpengvirus Cas4 protein. Maximum likelihood trees for a) Csf2 (Cas7), b) Cas4, c) CysH-like protein, d) Csf3 (Cas5), e) Csf1, and f) Csf4 (Cas11). Fuxivirus proteins are marked with red pentagrams. Kunpengvirus Cas4 protein is highlighted with blue in panel b).


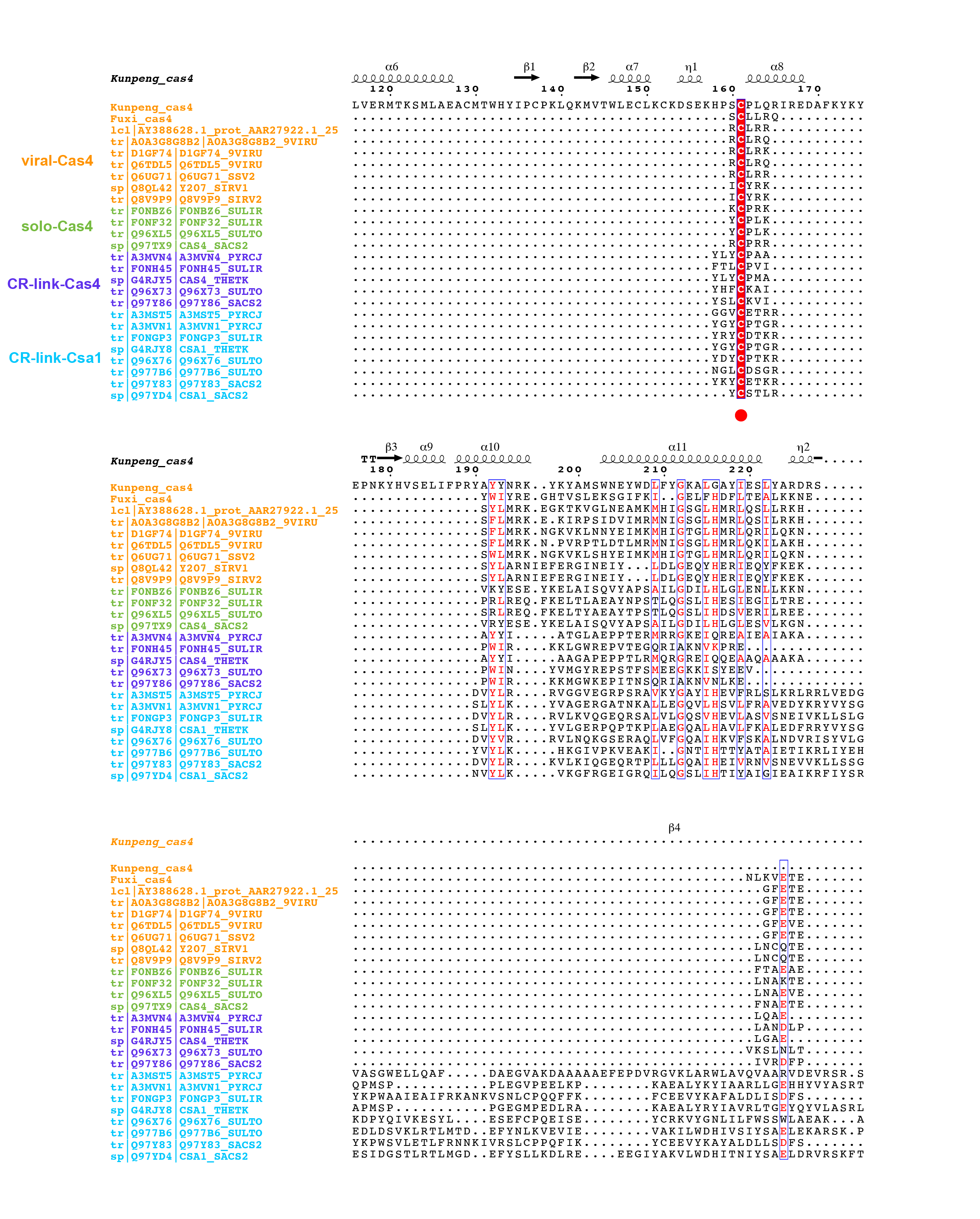

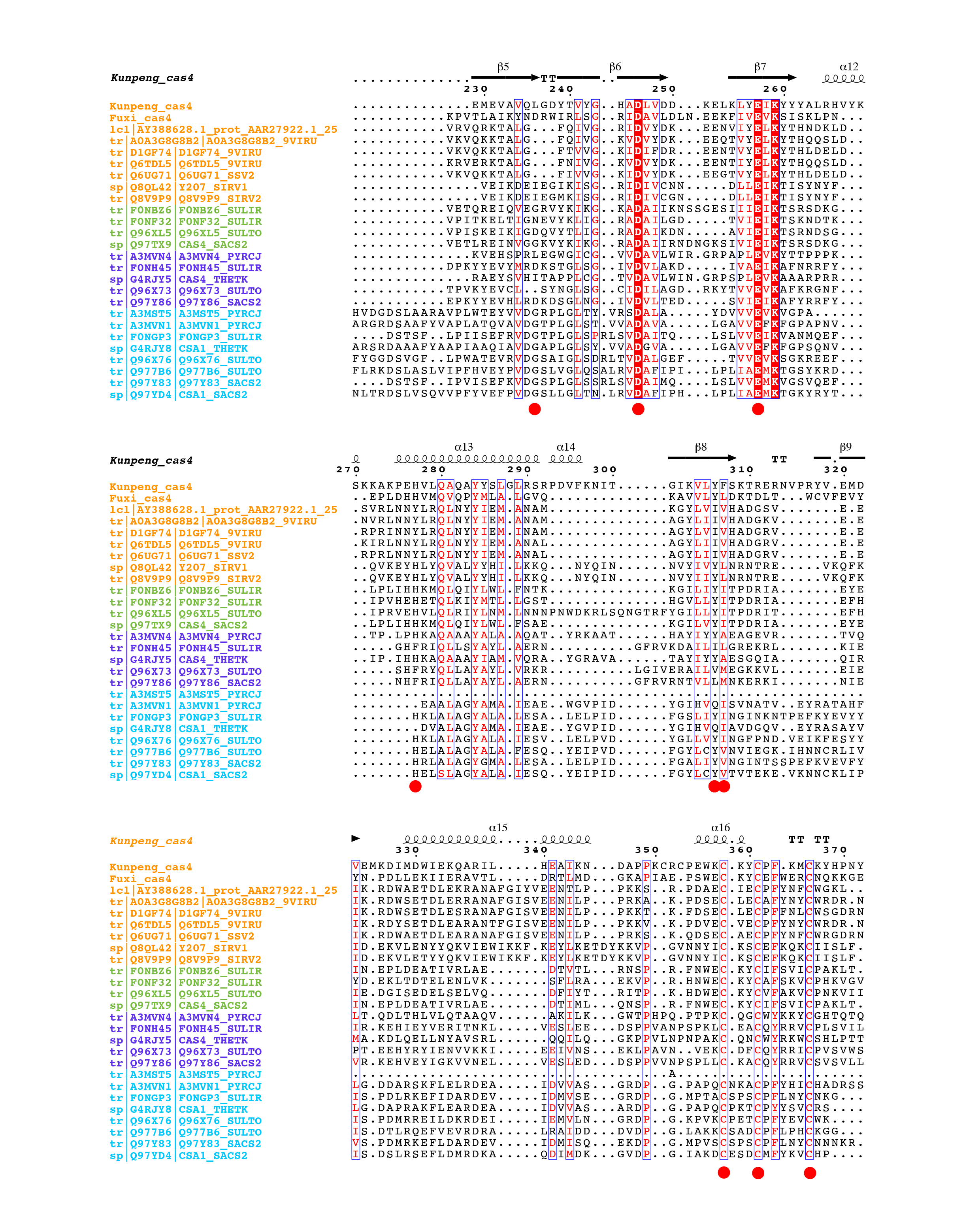


Extended Data Fig. S7: Sequences alignment of Cas4 protein. Conserved amino acid residues involved in the functions of CRISPR spacer acquisition are highlight in red dot.


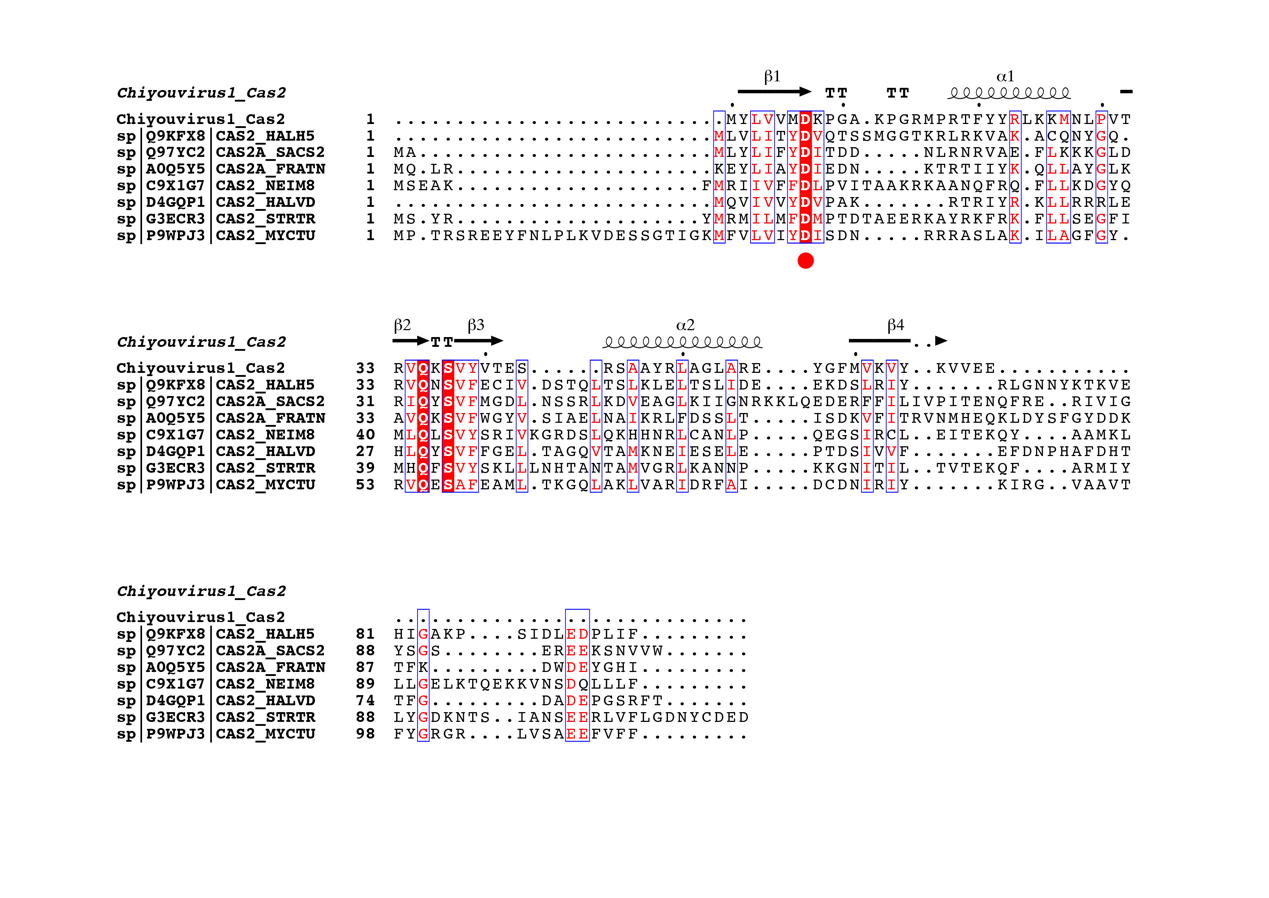


Extended Data Fig. S8: Sequences alignment of Cas2 protein. Conserved amino acid residues involved in Mg^2+^ binding site is highlight in red dot.


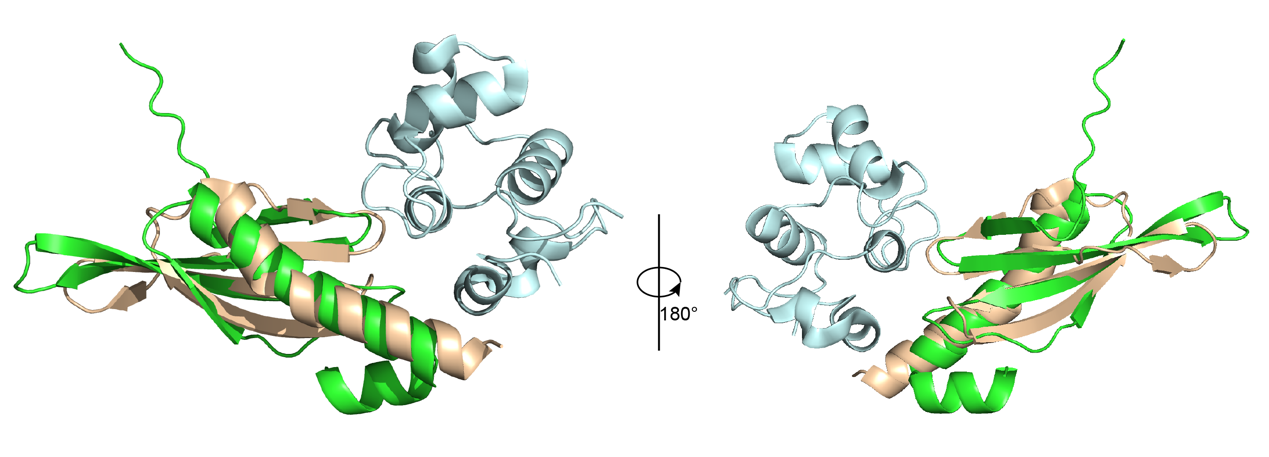


Extended Data Fig. S9: Structure alignment of predicted anti-CRISPR protein (Acr) of Chiyouvirus with reference AcrIF24. Models of Bathyarchaeia virus encoding Acr is colored in green, AcrIF24 NTD is colored in wheat. AcrIF24 CTD is colored in palegcyan.

**References**

1. Kim D, Park S, Chun J. Introducing EzAAI: a pipeline for high throughput calculations of prokaryotic average amino acid identity. *J. Microbiol.* **59**, 476-480 (2021).
